# Development and validation of a mixed-tissue oligonucleotide DNA microarray for Atlantic bluefin tuna, *Thunnus thynnus* (Linnaeus, 1758)

**DOI:** 10.1186/s12864-015-2208-7

**Published:** 2015-11-25

**Authors:** Željka Trumbić, Michaël Bekaert, John B. Taggart, James E. Bron, Karim Gharbi, Ivona Mladineo

**Affiliations:** University Department of Marine Studies, University of Split, Split, Croatia; Institute of Aquaculture, School of Natural Sciences, University of Stirling, Stirling, Scotland UK; Edinburgh Genomics, School of Biological Sciences, University of Edinburgh, Edinburgh, EH9 3FL Scotland UK; Institute of Oceanography and Fisheries, Split, Croatia

**Keywords:** *Thunnus thynnus*, transcriptome, Microarray, Tissue gene expression, genome mapping

## Abstract

**Background:**

The largest of the tuna species, Atlantic bluefin tuna (*Thunnus thynnus*), inhabits the North Atlantic Ocean and the Mediterranean Sea and is considered to be an endangered species, largely a consequence of overfishing. *T. thynnus* aquaculture, referred to as fattening or farming, is a capture based activity dependent on yearly renewal from the wild. Thus, the development of aquaculture practices independent of wild resources can provide an important contribution towards ensuring security and sustainability of this species in the longer-term. The development of such practices is today greatly assisted by large scale transcriptomic studies.

**Results:**

We have used pyrosequencing technology to sequence a mixed-tissue normalised cDNA library, derived from adult *T. thynnus*. A total of 976,904 raw sequence reads were assembled into 33,105 unique transcripts having a mean length of 893 bases and an N50 of 870. Of these, 33.4 % showed similarity to known proteins or gene transcripts and 86.6 % of them were matched to the congeneric Pacific bluefin tuna (*Thunnus orientalis*) genome, compared to 70.3 % for the more distantly related Nile tilapia (*Oreochromis niloticus*) genome. Transcript sequences were used to develop a novel 15 K Agilent oligonucleotide DNA microarray for *T. thynnus* and comparative tissue gene expression profiles were inferred for gill, heart, liver, ovaries and testes. Functional contrasts were strongest between gills and ovaries. Gills were particularly associated with immune system, signal transduction and cell communication, while ovaries displayed signatures of glycan biosynthesis, nucleotide metabolism, transcription, translation, replication and repair.

**Conclusions:**

Sequence data generated from a novel mixed-tissue *T. thynnus* cDNA library provide an important transcriptomic resource that can be further employed for study of various aspects of *T. thynnus* ecology and genomics, with strong applications in aquaculture. Tissue-specific gene expression profiles inferred through the use of novel oligo-microarray can serve in the design of new and more focused transcriptomic studies for future research of tuna physiology and assessment of the welfare in a production environment.

**Electronic supplementary material:**

The online version of this article (doi:10.1186/s12864-015-2208-7) contains supplementary material, which is available to authorized users.

## Background

Fish represent the oldest and most diverse vertebrate group, inhabiting every available aquatic environment [1]. They display remarkable heterogeneity of life trait characteristics and occupy a historically important place in human culture as a source of nutritionally valuable food. Marked by genome duplication events, the diversification and adaptive radiation of fish represent an attractive evolutionary model to study ecological, behavioural and physiological aspects of biodiversity [2]. Migratory tunas (family *Scombridae*, order *Perciformes*: suborder *Scombroidei*), are pelagic oceanodromous top predators, which display a set of extreme adaptations to life in a prey depauperate pelagic environment. They are obligate ram ventilators with streamlined bodies for rapid locomotion, have increased aerobic scope and metabolic rates that allow them to satisfy the energy needs of multiple high performance functions without compromise, including high digestive rates, increased somatic and gonadal growth and incredibly fast recovery from oxygen debt after exhaustive exercise compared to other teleosts [3, 4]. A key characteristic of tunas is their ability to retain metabolic heat in the brain, red muscle and viscera via vascular specialisations called *rete mirabile*, which comprise numerous juxtaposed arterial and venous vessels acting as a counter-current heat exchange system [4]. This heterothermic physiology shares characteristics of both poikilothermic and homeothermic organisms and is considered to have developed in order to permit thermal niche expansion, especially in correlation with maintenance of central nervous system function [5] or to sustain a rise in aerobic capacity and muscular performance [6].

The largest of the tuna species, Atlantic bluefin tuna, *Thunnus thynnus* (Linnaeus, 1758), inhabits the North Atlantic Ocean and the Mediterranean Sea. Based on the observed homing behaviour to two major spawning grounds, the Gulf of Mexico and the Mediterranean Sea [7], the International Commission for the Conservation of Atlantic Tunas (ICCAT) recognises a smaller Western and larger Eastern population as separate management units divided by the 45°W meridian. Although the differentiation between these populations has been corroborated by genetic data [8, 9], results of electronic tagging reveal a complex ecology, with extensive trans-Atlantic migrations and mixing of individuals at the foraging and spawning grounds [10, 11], complicating *T. thynnus* stock management. In high demand for sushi and sashimi preparation in the Japanese market, both populations of *T. thynnus* are considered to be depleted and overfished [12–14]. While latest assessments report a reduction in fishing mortality and increase in spawning stock biomass, allowing for moderate catch quotas increase after a long time in 2015 [15], these populations remain under threat. Capture-based tuna aquaculture, developed in the Mediterranean Sea over the past two decades, relies on yearly population renewal from the wild, fattening fish to market size in 3 months to 2 years, but is not thought to be sustainable in the long run [16]. Consequently, it is considered that only domestication of *T. thynnus* and development of high welfare aquaculture practices independent of wild resources can preserve the species for future generations. Extensive research efforts have been made regarding the control of *T. thynnus* reproduction in captivity [16, 17], a prerequisite for the development of a sustainable aquaculture industry. As evidenced from the Pacific bluefin tuna (*T. orientalis*) aquaculture development in Japan [18], the first bluefin tuna species successfully grown in captivity, a comprehensive approach will be required to solve many technical problems in order to meet this species’ physiological needs.

Large scale transcriptomic studies of important life trait characteristics, such as growth, maturation, environmental tolerance and disease resistance, can provide important knowledge concerning underlying molecular mechanisms and control elements, assisting in the development of welfare-centred and sustainable management strategies for aquaculture species, *e.g.* [19, 20]. With the increasing availability of high throughput sequencing technologies, development of the necessary tools and resources for broad-scale transcriptomics studies can be achieved in a relatively short period of time, even for species with a low level of background information [21]. Until recently, publicly available genomic resources for *T. thynnus* have been relatively scarce, most being derived from a single EST (expressed sequence tag) sequencing project comprising adult liver, ovary and testis mRNA transcripts contributed by Chini et al. [22]. This resource served as the basis for the construction of the first *T. thynnus*-specific oligonucleotide DNA microarray, which was employed for the description of the Gulf of Mexico *T. thynnus* gonad transcriptome [23]. It was also utilised to profile the cardiac transcriptome response to temperature acclimation in congeneric *T. orientalis* [24], highlighting the applicability of such a tool for gene expression analyses in phylogenetically related species. The release of a genome for *T. orientalis* [25] opens new opportunities for tuna comparative genome studies and ongoing transcriptome characterisation projects.

The objectives of the current study were: 1) to contribute to and expand existing transcriptomics resources for studying *T. thynnus*, 2) to use the newly developed resource to design and validate a custom oligo-microarray, and 3) to employ the newly developed microarray to investigate functional differences in transcript expression among tissues. To this end, pyrosequencing technology was used to sequence adult mixed-tissue normalised cDNA library providing the template for the development of a custom 8 × 15 K Agilent oligo-microarray. The microarray was used to compare: gill, heart, liver, ovaries and testes of adult fish and to infer their respective transcriptional specificities. The derived transcriptome was also compared to *T. orientalis* genome.

## Methods

### Animal tissue collection and RNA extraction

All fish handling procedures were conducted in accordance with established standards for the care and use of animals of the Ethical committee for animal welfare at the Institute of Oceanography and Fisheries, Croatia. No specific permits were required as samples were collected during standard commercial operations without any form of experimental manipulation.

Atlantic bluefin tuna were reared in mid-Croatian offshore farm cages for two years. During the winter harvest of 2012, adult fish (curved fork length (mean ± SD) = 177 ± 9 cm; weight = 99 ± 14 kg) were sacrificed by pithing and samples of kidney, spleen, liver, gonads, gills, small intestine, heart (*apex ventriculi*), red and white skeletal muscle, skin scrapes and whole blood, were dissected and preserved in RNA*later* stabilisation solution (Qiagen, UK) at −20 °C, after overnight storage at +4 °C. In addition, liver and kidney samples from juvenile moribund fish with signs of septicaemia were available from another study [26] and were included in a normalised cDNA library preparation to increase the diversity of the sequenced transcriptome.

Between 30 – 40 mg of each of the tissues were homogenised separately in 1 mL of TRI Reagent (Sigma-Aldrich, UK) by 2 × 35 s disruption runs on a Mini-Beadbeater-24 (BioSpec Products, Inc, USA) and total RNA was extracted according to manufacturer’s instructions. The RNA precipitation step was modified to include a high-salt solution as recommended for polysaccharide- and proteoglycan-rich sources [27]. Integrity of total RNA preparations was assessed by ethidium bromide agarose gel electrophoresis and purity and concentration by spectrophotometry (NanoDrop ND-1000, Thermo Scientific, USA).

### Normalised cDNA library preparation and sequencing

A mixed RNA sample comprising equal amounts (5 μg) derived from all sampled tissues, except ovaries, was created from one female, one male and one moribund juvenile specimen and purified using RNeasy columns (Qiagen, UK). Because *T. thynnus* ovaries showed a significantly increased proportion of the small RNA fraction comprising tRNA and 5S RNA, as previously found in amphibians [28, 29] and several other teleosts [30, 31], poly(A) + RNA was first isolated from the ovarian total RNA population using a Poly(A) Purist kit (Ambion, UK) and subsequently 200 ng of the isolated mRNA were included in the pool. For normalisation, the MINT kit (Evrogen, Russia) was initially used to construct a full-length-enriched double stranded cDNA library from 1.5 μg of the tissue RNA mixture, according to manufacturer’s protocol. The TRIMMER kit (Evrogen, Russia) was used to normalise 1 μg of cDNA starting material, applying the duplex-specific nuclease (DSN) method [32]. The final normalised and amplified cDNA library was purified using a MinElute PCR Purification Kit (Qiagen, UK) and ranged in size from 0.25 to 3 kb, as visualised on a 1.5 % agarose/ethidium bromide gel.

GS FLX Titanium library preparation and sequencing were performed by the Edinburgh Genomics facility (University of Edinburgh, UK). Approximately 5 μg of normalised cDNA were used for generating a 454 sequencing library using the GS FLX Titanium Rapid Library Preparation kit (Roche Applied Science, UK), following manufacturer’s instructions. Sequencing was performed using The Genome Sequencer™ (GS) Titanium FLX instrument (Roche Applied Science, UK). Bases were called with 454 software and reads trimmed to remove adapter sequences.

### Data filtering and assemblies

Low-quality reads (Phred score < 30) were filtered out and PRINSEQ v0.20 [33] was used to remove PCR duplicates and low complexity sequences. Two complementary sequence read assembly methods were chosen for this study: WGS-assembler 7.0 [34] and Mosaik-aligner v2.1 [35] to assemble the contigs based on the Genbank archived *T. thynnus* EST database [36]. Subsequently, to lower the redundancy resulting from *de-novo* assemblies, TRF 4.07b [37] was used to remove all transcripts shorter than 500 bases or exhibiting repetition, with entropy above 1.

### Sequence annotation and functional assignments

The longest coding DNA sequences were determined for each transcript using the command-line program getorf from the EMBOSS v6.6.0 package [38]. ESTScan v2 [39] was then used to confirm transcript coding regions and determine sequence orientation. The BLAST algorithm [40] was used for sequence similarity searches against public databases. The coding sequences of the predicted transcripts were annotated using BLASTP searches against the GenBank Reference Proteins database [41], with an expectation value (e-value) cut-off of 10^−4^, minimum similarity of at least 60 %, and minimum alignment length of 33 amino acids. Additionally, the transcripts were annotated using BLASTN searches against the annotated EST *T. thynnus* database [36]. The inferred annotations were used to retrieve Gene Ontology (GO) annotation for molecular function, biological process and cellular component [42]. To avoid redundant functional assignments, the best-rated similarity hit with at least one GO annotation was chosen. A custom pipeline converted GO terms to GO Slim terms, using the Protein Information resource and Generic GO Slim files [43]. Kyoto Enyclopedia of Genes and Genomes (KEGG) database [44] was used to infer functional annotation of the sequences through KAAS (KEGG Automatic Annotation Server) [45] using single-directional best hit method for ESTs. Sequences with assigned KO (KEGG Orthology) identifiers were categorised into functional groups according to the KEGG BRITE hierarchy, excluding human diseases.

### Genome mapping

Generated sequence data was compared with two perciform fish genomes that have been publicly released: *Thunnus orientalis* (Pacific bluefin tuna; NCBI Assembly GCA_000418415.1;[25]), and *Oreochromis niloticus* (Nile tilapia, NCBI Assembly GCA_000188235.2). The 133,062 contigs (684,497,465 bp) of *T. orientalis* and the 77,755 (927,696,114 bp) of *O. niloticus* genomes were downloaded and BLASTN [40] was used to search for similar transcripts. Default parameters were used for BLASTN searches, with the following exceptions to account for the divergence and short length of the sequences available: minimum alignment size 80 nt, minimum percentage of sequence identity 25 %, maximum e-value 0.001 and low complexity mask on. Results were displayed using a hive plot [46].

### *T. thynnus* microarray design

Transcripts with a significant match to a reference protein were chosen for microarray probe design. In addition, 57 publicly available full mRNA sequences from the *T. thynnus* nucleotide (NCBI) database were also included. Sixty-mer oligonucleotide probes were designed using the eArray (Agilent Technologies, UK) online probe design tool with Base Composition and Best Probe Methodologies, 3’bias and sense orientation. Two probes were designed for each target transcript. After initial screening, unique probes showing no cross-hybridisation potential were selected to produce an 8 × 15 K Agilent custom oligo-DNA microarray design format (Agilent Design ID = 038391), comprising 15,208 user defined features and 536 Agilent positive and negative controls. Target transcripts (*N* = 6,439) with best annotation were represented with two probes on the array and the remainder (*N* = 2,190) with a single probe. In all, 6,287 unique proteins were annotated. For calculation of the multiplicative detrending step implemented within the Agilent Feature extraction software 35 probes were replicated five times.

### Microarray experiment

For microarray validation, five metabolically distinct tissues were chosen from adult *T. thynnus*: gill, heart, liver, testes and ovaries (4 biological replicates per tissue). A total of 20 arrays (4 replicates x 5 tissues) were assayed in the final common reference pool design, with the pool comprising a contribution from each biological sample. Amplified and fluorescently labelled cRNA (complimentary RNA) for microarray hybridisations was prepared in accordance with the Two-Color Microarray-Based Gene Expression Analysis - Low Input Quick Amp Labelling Protocol v6.6 (Agilent Technologies, USA). The cDNA synthesis was initiated with 150 ng of total RNA from each tissue sample, this being primed with an Oligo dT – T7 RNA polymerase promoter. Subsequently, T7 RNA polymerase incorporated Cyanine 3 (Cy3) or Cyanine 5 (Cy5) – CTPs into a growing chain of antisense cRNA. Amplified cRNA was purified using the GeneJET RNA Purification Kit (Thermo Scientific, UK) following the manufacturer’s RNA Cleanup Protocol and was further quality-checked by spectrophotometry (NanoDrop ND-1000, Thermo Scientific, USA) and agarose gel electrophoresis. Following amplification and purification, 300 ng of each Cy3 labelled test and 300 ng of Cy5 labelled reference pool cRNA were mixed together and incubated with Fragmentation and Hybridization buffers according to manufacturer’s instructions. Competitive hybridisations were carried out in a rotary oven (Agilent Technologies, UK) over 17 h at 65 °C and 10 rpm. Following Agilent standard protocols, the slides were subsequently washed in Wash Buffer 1, Wash Buffer 2, acetonitrile and Agilent Stabilization and Drying solution, the latter to reduce ozone-induced decay of Cy5 signal. Slides were stored in light proof box and scanned within 1 h of washing.

### Microarray data analyses

Scanning was conducted using an Axon Genepix 4200A scanner with Genepix Pro 6.1 image acquisition software (Molecular Devices, UK) with 60 % red and 90 % green laser power at 5 μm resolution. Saturation tolerance was set to 0.05 % and automatic photo-multiplier tube (auto-PMT) gain used to achieve similar mean intensities of Cy3 and Cy5 signals. Acquired data were exported and processed using the Agilent Feature Extraction software (v9.5.3.1) to obtain background-subtracted signals, as well as other spot statistics and quality metrics. Scan data were analysed using GeneSpring GX v12 (Agilent Technologies, UK). Baseline transformation was not employed and data were normalised using a Lowess model. Principal component analysis (PCA) was conducted to visually inspect the distribution of gene expression variance among arrays within and between experimental conditions. Following removal of Agilent control features, stringent quality filtering involved removal of saturated and non-uniform features, population outliers and features that were not significantly positive with respect to the local background.

One-way ANOVA unequal variance (Welch) (*p* < 0.05) was applied to preselect features showing potential differences in gene expression between tissues. Unsupervised network analysis was conducted on identified features using Biolayout *Express*^3D^ [47], using a Pearson coefficient of correlation as a similarity measure and threshold over 0.94. The network graph was clustered into distinct patterns of gene expression using a Markov clustering algorithm (MCL) with inflation value set to 2.0 and smallest cluster allowed being 10, to achieve optimal granularity of clusters and focus only on larger areas of high connectivity [47].

Functional gene set analyses were performed on the entire list of quality filtered entities based on KEGG KO identifiers. During the analyses, gene expression profiles were collapsed into 3,222 unique KOs using the median method. Tests were run to identify significantly up- and down- regulated pathways for each tissue and their pairwise combinations against all other samples using Generally Applicable Gene-set Enrichment (GAGE) analyses [48], implemented as R/Bioconductor package [49], which is robust to small datasets with different sample sizes. A default FDR q-value of 0.1 was used as a cut-off. Pathways were subsequently grouped into KEGG functional categories and the difference between the counts of up- and down- regulated pathways in each category hierarchically clustered and displayed as a heatmap.

### RT-qPCR validation

Ten transcripts from different Biolayout derived clusters showing tissue-specific and diverse expression profiles, and spanning a wide range of fold change (FC) values, were chosen for RT-qPCR validation. Three additional genes were tested to serve as internal controls (reference genes): elongation factor-1α (*elf-1α*) [50], beta-actin (*actb*) [26] and a transcript showing a stable expression profile across the experimental conditions, annotated as FtsJ methyltransferase domain containing 2 (*ftsjd2*), from the microarray. Primer3 [51] software was used to design primers for the selected sequences with at least one in the pair overlapping the target hybridisation area of the microarray 60-mer probe. Complementary DNA (cDNA) was synthesised from 2 μg of the same total RNA extractions used for the microarray experiment employing the High Capacity cDNA Reverse Transcription Kit (Applied Biosystems, UK). The synthesis was primed with 1.5 μl of random hexamer primers supplied with the kit and 2.5 μM anchored-oligo (dT)_20_ (Eurofins, Germany) per 20 μl reaction. DNase treatment was not performed prior to cDNA synthesis as it may result in RNA degradation [52]. Instead, minus RT (RT-) controls (reverse transcriptase replaced with water) were created to control for the presence of genomic DNA in the extractions.

Real-time PCR assays (20 μl) were performed on a Mastercycler ep realplex2 (Eppendorf, Germany) using Luminaris Color HiGreen qPCR Master Mix (Thermo Scientific, UK), in duplicate with 25 × cDNA template dilutions and 0.3 μM of each primer, according to the following thermal profile: 50 °C for 2 min (Uracil-DNA Glycosylase pre-treatment), 95 °C for 10 min, followed by 40 cycles of denaturation at 95 °C for 15 s, annealing at 60 °C for 30 s and elongation at 72 °C for 30 s. After amplification a melting curve analysis was performed from 55 °C to 95 °C at 0.5 °C increments for 15 s each to verify single product amplification. The presence of the correct size of PCR product was also verified by agarose gel electrophoresis. Six-point standard curves of a 4 × dilution series of a pool of all starting cDNAs were run with every assay to determine primer-specific efficiencies. A standard curve was used to extrapolate non-normalised relative copy numbers for each gene and the BestKeeper application [53] was used to inspect the suitability of reference genes for normalisation.

To compare the fit between qPCR and microarray fold changes (FCs), we calculated the concordance correlation coefficient (CCC) as well as other regression metrics previously described by Miron *et al.* [54]. The statistical significance of individual targets’ expression profiles was examined on the combined qPCR and microarray data for each gene, coded by their respective percentile ranks, using a nonparametric Kruskal-Wallis test implemented in R software.

## Results and discussion

### Pyrosequencing and transcriptome assembly

A total of 976,904 raw sequence reads were generated, with a mean size of 347 bases (b) (Table 1), which is consistent with the sequencing technology used [55]. The reads that passed quality control filtering were trimmed of sequencing primers and adaptors and used for the assembly process. WGS-assembler (Celera) generated a *de-novo* transcriptome assembly of 70,108 contigs and 52,452 singletons (Table 1). Contigs and singletons were scaffolded using Mosaik based on the 10,163 available ESTs. Based on the high quality reads of over 500 b and of higher complexity, 33,105 unique transcripts were assembled, with a mean length of 893 b and an N50 of 870. Of these, 22.0 % of the transcripts had a length more than 1,000 b with 22.8 % of these having a full-length coding region. To evaluate the quality of the assembled transcripts, all the usable sequencing reads were realigned to the transcripts.

**Table 1:** Statistics of the assembled *T. thynnus* sequences

Raw data	
Number of reads	976,904
Mean read size	347
Total size of reads	338,522,590
After assembly (no singletons)	
Number of transcripts	70,108
Mean size	593
Total size of transcripts	41,564,882
After assembly (singletons only)	
Number of transcripts	52,452
Mean size	431
Total size of transcripts	22,601,482
After filtering (>500 b)	
Number of transcripts	33,105
N50	870
Total size of transcripts	29,512,631
Shortest transcript	500
Longest transcript	4,955
Mean size	893
Median size	799
Mean GC%	43.5 %
N%	0.2 %

### Functional inference by searching against public databases

The assembled transcripts were annotated using BLASTP and BLASTN searches against the RefSeq Proteins [41] and EST databases respectively [36]. The results indicated that out of 33,105 transcripts, 11,065 (33.4 %) showed significant similarity to known proteins or gene transcripts in the RefSeq Proteins database. To evaluate the *T. thynnus* coverage of the assembled transcripts, the 10,163 EST available for *T. thynnus* were aligned to the 33,105 transcript sequences generated in this study and vice versa. In total, 4,939 (48.6 %) of all *T. thynnus* ESTs aligned to at least one transcript, whereas 4,394 (13.3 %) of the new transcripts had at least one corresponding *T. thynnus* EST. This demonstrates that the strategy employed captured some of the existing data [22] and added new information to *T. thynnus* database.

**Fig. 1:**
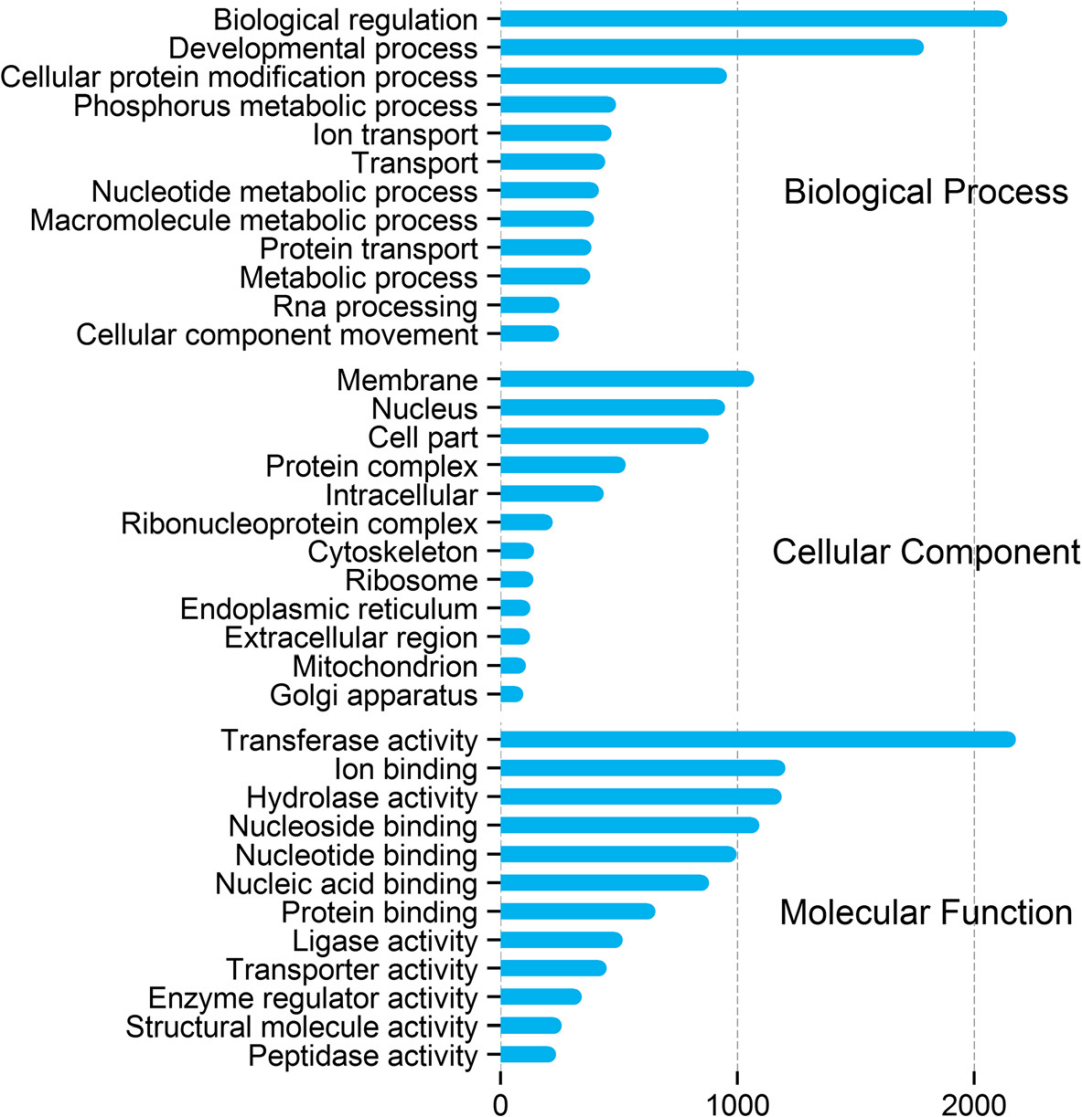
Multilevel Gene Ontology categorisation of the annotated *T. thynnus* transcripts. GO annotations were first converted to GO-Slim annotations and the multilevel chart shows the top twelve of each category to reduce the complexity of the chart. Bar length reflects the total number of GO term hits recorded for a given category across the dataset

**Fig. 2:**
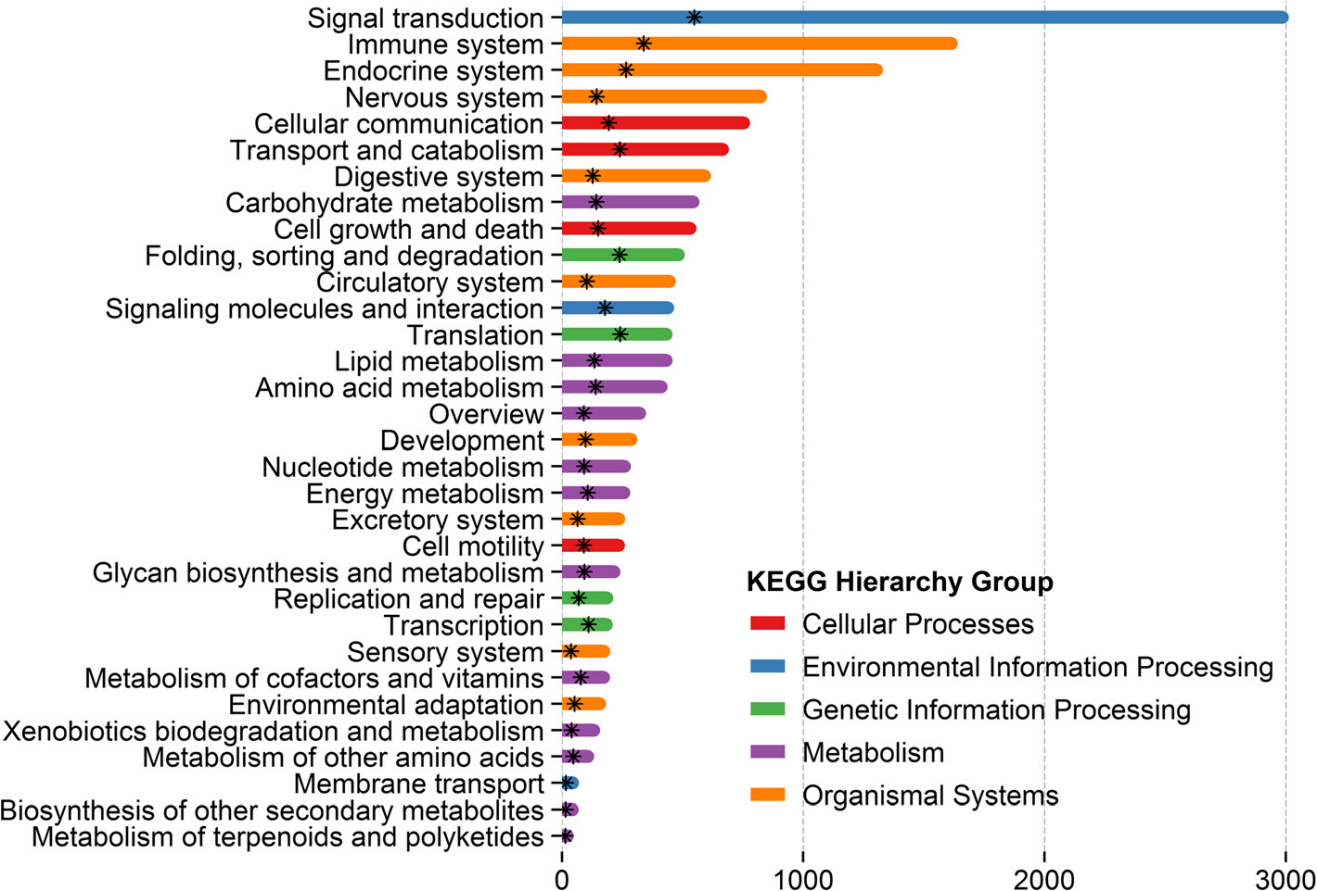
KEGG functional annotation of *T. thynnus* transcripts. The pathways were categorised based on BRITE hierarchy functional groups excluding human diseases. Bars represent total count of KEGG annotated transcripts per group. The star marks the number of unique KOs per functional group

GO functional annotation was assigned to the assembled *T. thynnus* transcripts/genes on the basis of RefSeq Proteins annotation. Out of 11,065 transcripts that had similarity to known gene products, 8,384 (25.3 % of total library) were assigned a GO annotation. Figure 1 shows that derived GO descriptions for *T. thynnus* transcripts cover a diverse set of molecular functions, cellular components and biological processes. Similarly, 8,334 (25.2 %) out of the total 33,105 transcripts were assigned 4040 unique KO identifiers and mapped to 262 different biochemical pathways in the KEGG BRITE functional hierarchy, excluding human diseases. Pathways were categorised according to their functional groups and total transcript abundance with unique KO identifiers found in each group is outlined in Fig. 2. The top five best represented categories were signal transduction, immune, endocrine, nervous system and cellular communication. Figure 2 also points to a substantial level of functional redundancy, as evident from lower number of unique KOs found in each group. This is an inherent feature of the KEGG classification as many pathways share the same genes and some KOs are also assigned to different transcripts with similar functional motifs.

### Genome mapping

**Fig. 3:**
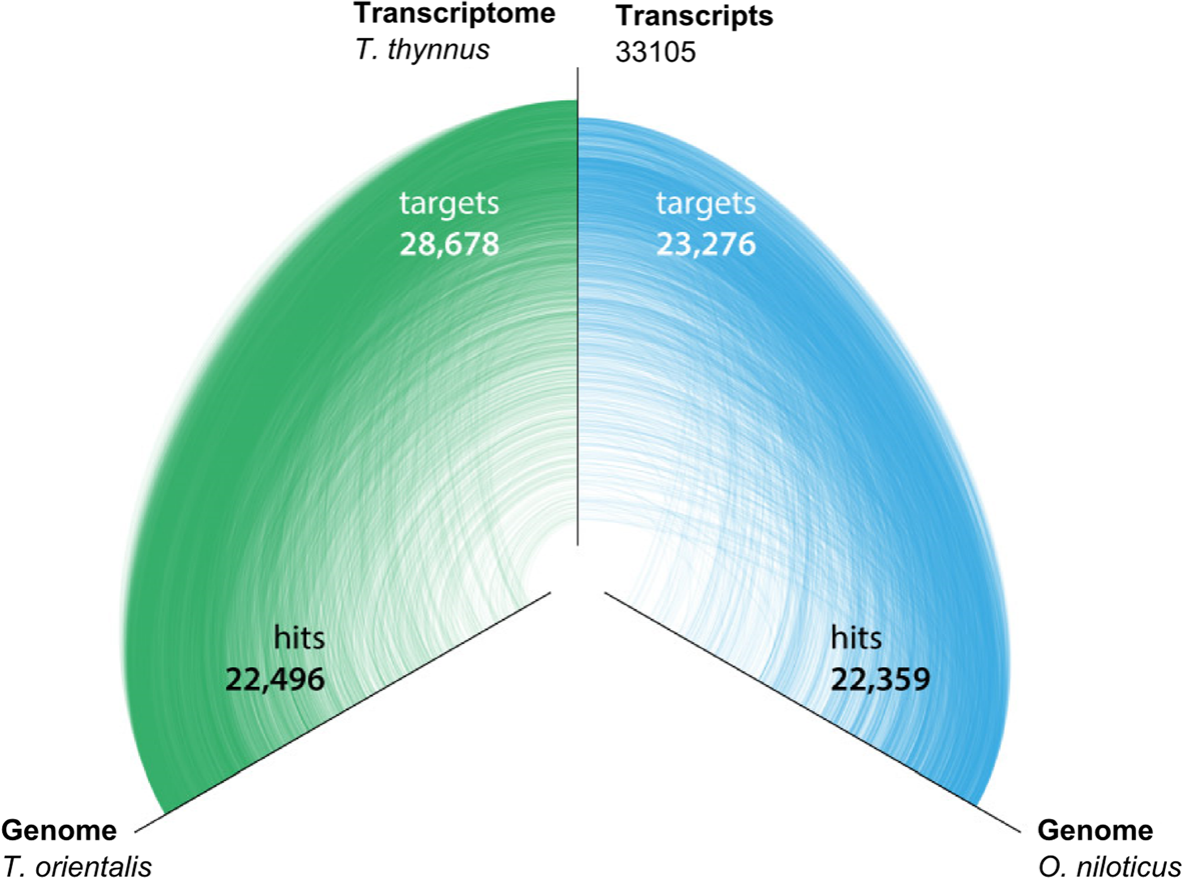
Mapping of *T. thynnus* transcripts to *T. orientalis* and *O. niloticus* genomes. Hive plot of the BLASTN results. The number of transcripts (target) with a similar region (hit) is reported for each genome

To assess the similarity of *T. thynnus* transcripts to currently available genomic resources, mapping was performed to two perciform genomic sequences: *T. orientalis* and *O. niloticus* (see Additional files 1 and 2). The genome assembly of *O. niloticus* is in a more advanced state, contigs being organised into linkage groups or chromosomes, while the *T. orientalis* genome is in its initial assembly release with sequences organised into contigs. The results of the mapping to the two genomes were visualised using a hive plot where each genome is placed along a linear axis and hits are drawn as curved links connecting *T. thynnus* transcripts to respective target regions in each genome (Fig. 3). As expected, more targets found a hit with the congeneric *T. orientalis* genome (28,678 or 86.6 %) than the more distantly related *O. niloticus* genome (23,276 or 70.3 %). *T. thynnus* and *T. orientalis* are considered to be very closely related and have been separated as sub-species until recently when molecular data, especially mitochondrial DNA, corroborated their separate species status [4, 56]. Our data also demonstrate their close connection and sequence similarity which makes the *T. orientalis* genome an important resource for *T. thynnus* studies as well, with possible applications for exon-intron gene modelling or reference mapping in future sequencing studies. The fraction of *T. thynnus* targets that did not find a match to *T. orientalis* genome consisted mainly of non-annotated singletons, with this group also showing increased repetitive nucleotide stretches (data not shown). These sequences could be difficult to match, or may represent *T. thynnus*-specific diversification, or both.

### Results of microarray transcriptional inference and qPCR validation

**Fig. 4:**
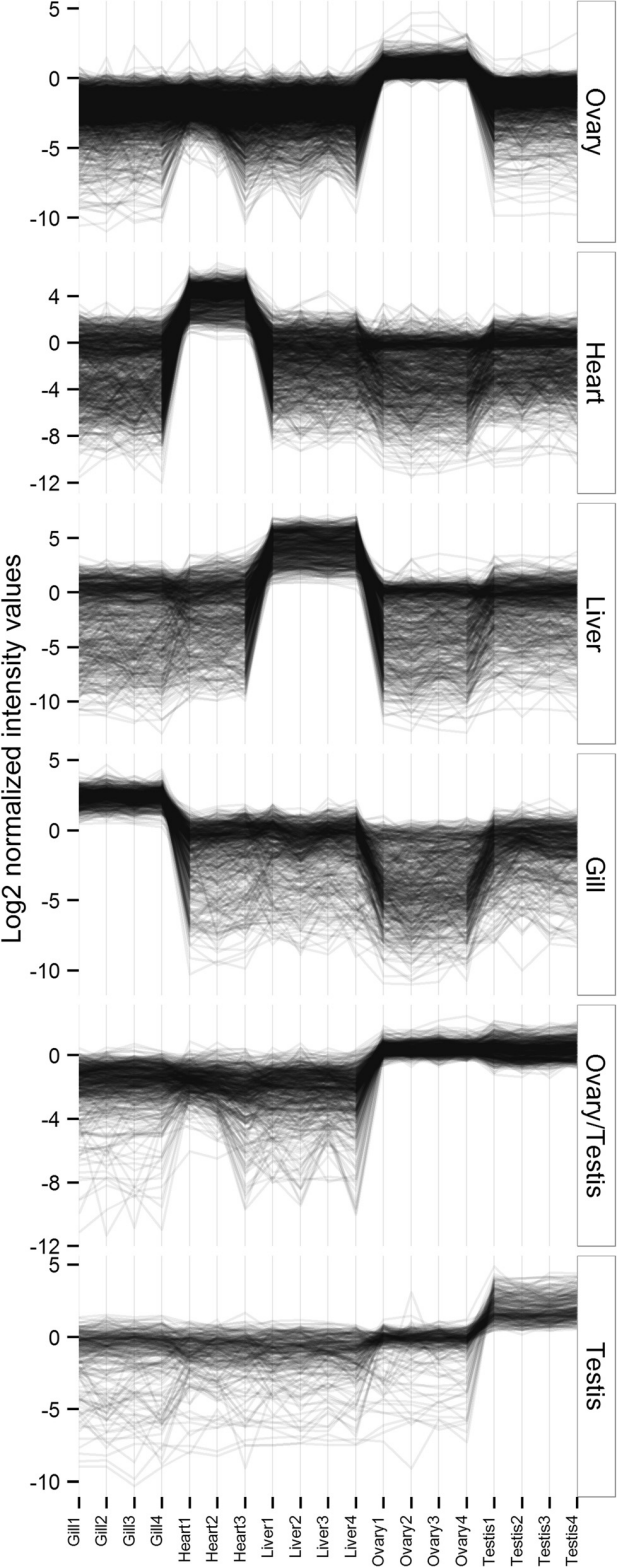
Clusters of tissue-specific gene expression profiles of adult captive *T. thynnus.* Clusters were derived using network analyses and Markov clustering algorithm in Biolayout *Express* ^3D^, plotted in descending order according to number of transcripts in cluster

Principal component analysis showed distinct separation of arrays into tissue-specific groups (data not shown). The signals from one array deviated considerably from its biological replicates within the heart group, reflecting technical difficulties noted during hybridisation, and therefore this array was excluded from further analyses. From 15,068 *T. thynnus*-specific probes, 13,494 passed the quality filtering across the experiment and showed positive and significant signals above background in both channels. Most of these features, 12,306 (91.2 %), were found to be differentially expressed (ANOVA, *p* < 0.05) between at least two tissues, consistent with the expected low number of genes ubiquitously and constitutively expressed in an organism [57]. Network analyses based on these preselected features produced a complex graph with 8,836 nodes, further reduced into 87 clusters of discrete patterns of gene expression (see Additional file 3). Over half of the nodes were grouped within the first six clusters (Table 2), each corresponding to one tissue-specific up-regulated group of features, and an ovary and testis combination (Fig. 4). The largest was an ovary-specific cluster while highest intensity values of expression were recorded for liver and heart transcripts. Other clusters not shown in Fig. 4 comprised entities with varying expression profiles across tissues.

**Table 2:** Size and tissue specificity of the first six gene expression clusters of *T. thynnus* obtained by MCL algorithm in Biolayout *Express* ^3D^

MCL Clusters	Tissue specificity	N (transcripts)
Cluster 1	Ovary	1,598
Cluster 2	Heart	877
Cluster 3	Liver	654
Cluster 4	Gill	579
Cluster 5	Ovary/Testis	557
Cluster 6	Testis	253
Other	Mixed	2,018
No Class	/	2,300
Grand Total		8,836

Eight transcripts representative of the six largest Biolayout clusters were chosen for the purpose of qPCR validation, taking into account their functional relevance and FC span (see Additional file 4 for details): aquaporin 3b *aqp3b* (Gill), sarcoplasmic reticulum calcium-ATPase 2 *atp2a2a* (Heart), complement C4-like *c4*, coagulation factor V *f5* (Liver), exosome component 2 *exosc2,* pescadillo *pes* (Ovary), somatolactin *sl* (Testis) and mediator complex subunit 6 *med6* (Ovary/Testis). Transcripts specific to particular tissue clusters show high expression in only that tissue, such that examination of expression gives low values for all other tissues. Thus two additional transcripts, biliverdin reductase B *blvrb* and prohibitin 2 *phb2,* were chosen as exemplars showing expression across a range of tissues. This ensured that the microarray validation did not comprise only tissue specific transcripts. However, as functional interpretation of results focuses on tissue-specific signatures, these two transcripts are not specifically discussed. Three reference genes were tested, *actb, elf-1α,* previously used in *T. thynnus* qPCR experiments [23, 26, 50], and a candidate from this microarray study, *ftsjd2*, which subsequently did not show stable expression profiles according to BestKeeper tool, exceeding the recommended quantification cycle (Cq) standard deviation of one [53]. This was not unexpected as we were comparing different tissues of adult fish; however, the variation was systematically attributed only to ovarian tissue, which was characterised by a very different total RNA population. Hence, non-normalised (normalised to total RNA used for cDNA synthesis) and normalised data (normalised to geometric mean of *actb*, *elf-1α* and *ftsjd2* = TEF) were further compared to microarray results. For all information pertinent to RT-qPCR validation of microarray results see Additional file 4.

**Fig. 5:**
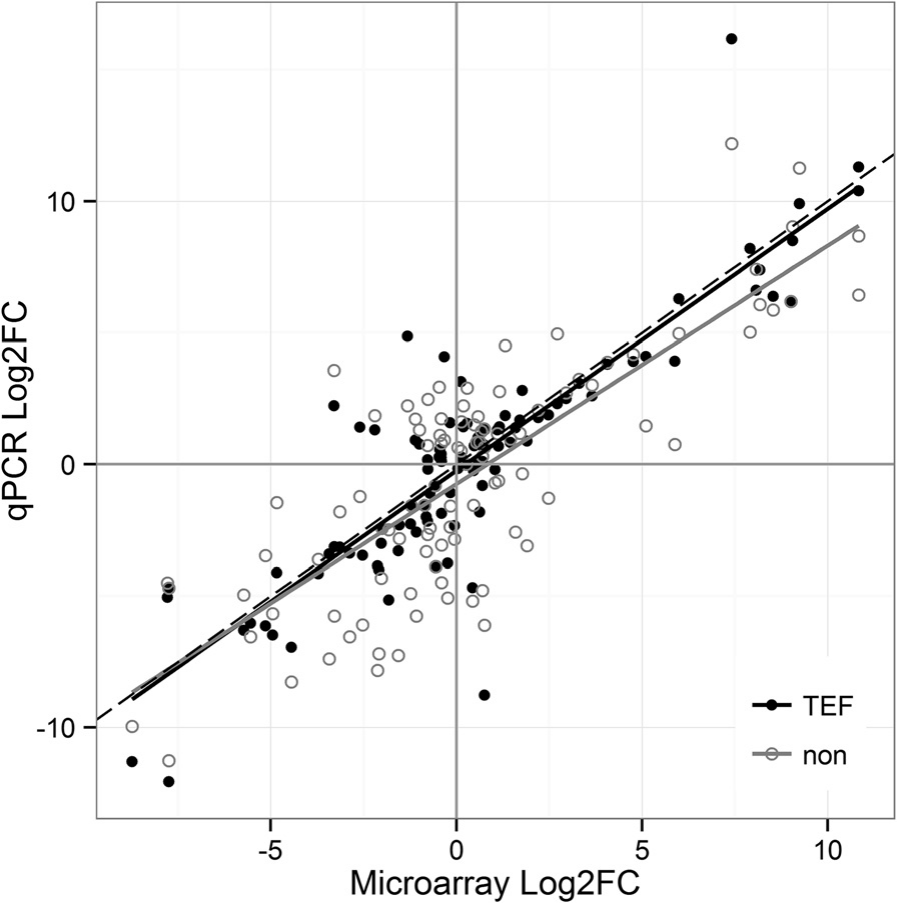
Least-squares regression fit between microarray and qPCR data. The fit between microarray and qPCR non-normalised (non) and normalised (TEF) Log2FC (Fold Change) is shown in respect to the identity line (dashed) for the selected target genes of *T. thynnus*

Microarray and qPCR fold changes (FCs) among all five tissues for ten target genes were compared in Log2 space using the concordance correlation coefficient (CCC), proposed for global validation of microarray experiments [54]. CCC is a product of a measure of precision (Pearson r), describing linear correlation of the data, and accuracy, how close the least-squares regression line is to the identity line. It takes values from −1 (perfect inverse agreement) to 1 (perfect agreement). We evidenced a good agreement between microarray and qPCR data in this study, both in terms of precision and accuracy (Table 3, Fig. 5). Due to the range enhancement artefact, Miron *et al*. [54] suggested that up- and down-regulated genes should be inspected separately. Gene expression profiles were broken into positive and negative FCs, with former producing better scores (Table 3), which is sometimes observed in microarray and qPCR comparisons [58]. Correlation of microarray and qPCR data is often affected by pitfalls and differences inherent to each technology, *i.e.* probe/primer properties and amplification kinetics, which can differ from one transcript to another and equally depend on its level of expression [52, 58].

**Table 3:** Agreement between qPCR and microarray data

	ALL DATA		UP		DOWN	
Indice	non	TEF	non	TEF	non	TEF
Slope	0.91	0.99	0.88	1.05	0.82	1.05
Y intercept	−0.75	−0.24	−0.54	−0.59	−1.08	0.05
Precision (r)^a^	0.80	0.87	0.77	0.85	0.54	0.73
Accuracy	0.98	0.99	0.96	0.97	0.89	0.94
CCC	0.78	0.86	0.73	0.82	0.48	0.69

Correlation correspondence index (CCC) and other indices describing agreement of microarray and qPCR data (non-normalised (non) and normalised (TEF)) are shown. Log2FCs (Fold Changes) were examined for all data and positively and negatively regulated values separately

^a^Correlation is significant at the 0.01 level (2-tailed)

**Fig. 6:**
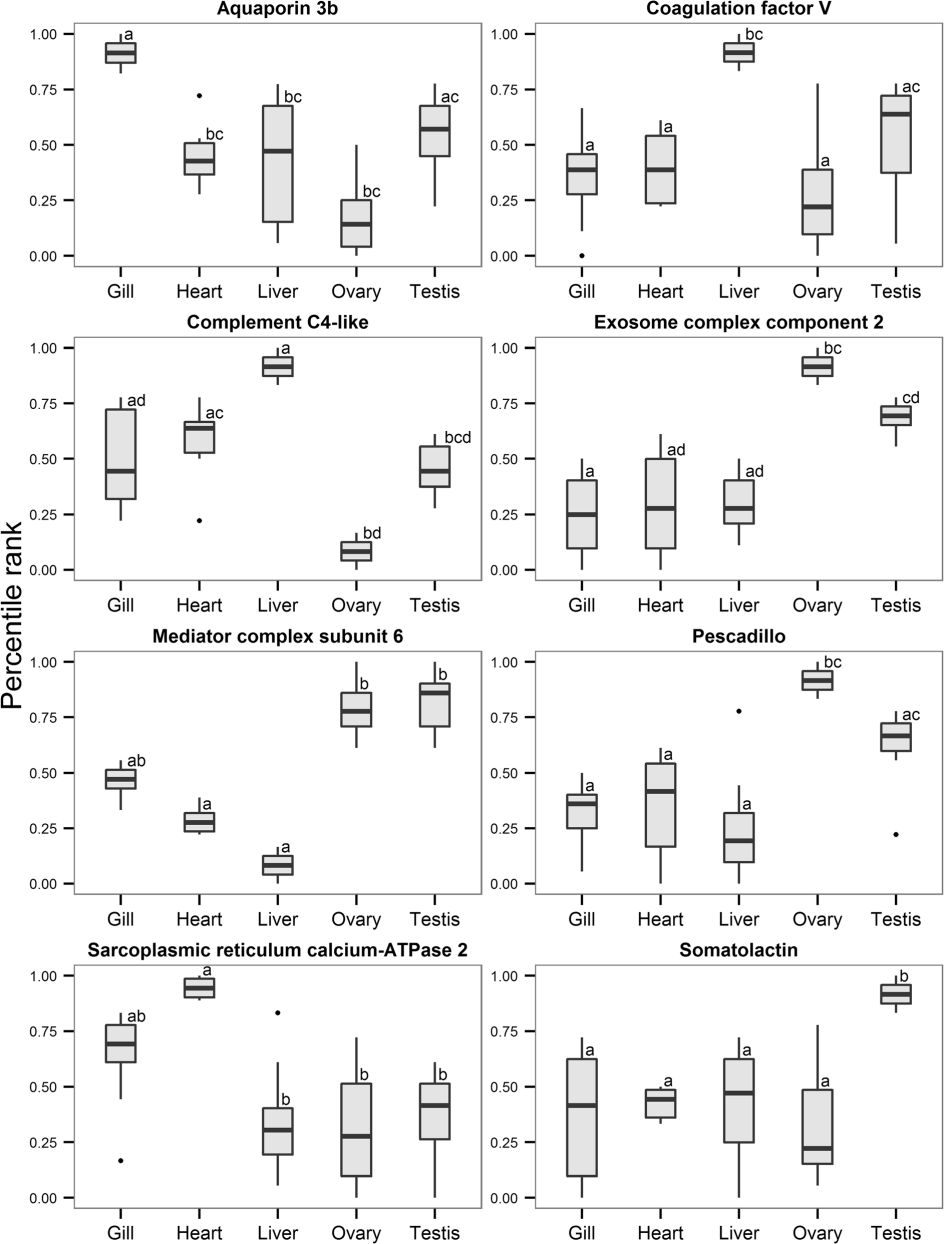
Combined expression profiles of target genes belonging to six largest *T. thynnus* tissue clusters. The profiles were derived by combining qPCR and microarray data for specific genes using their respective percentile ranks. Nonparametric Kruskal-Wallis test was used to determine statistical significance of the differences between tissues. Letter codes denote statistical significance at *p*< 0.05

As there was better concurrence between normalised qPCR and microarray data with respect to non-normalised, only the normalised set was included in the joint qPCR and microarray statistical analyses, with data coded by respective percentile ranks. The goal was to maximise power to test differences between tissues by combining two outputs and increasing the number of replicates for each condition. Figure 6 shows derived expression profiles for target transcripts representatives of the six largest Biolayout clusters. Results of post-hoc tests for all transcripts (*p* < 0.05) are found in Additional file 5. Although there was some disagreement between qPCR and microarray data evidenced by increased interquartile ranges in certain instances (Fig. 6), it is evident that selected transcripts exhibit highest expression in the tissue clusters to which they were assigned. However, this was not found to be statistically significant in all comparisons made (Fig. 6).

### Functional interpretation of tissue-specific expression profiles

**Fig. 7:**
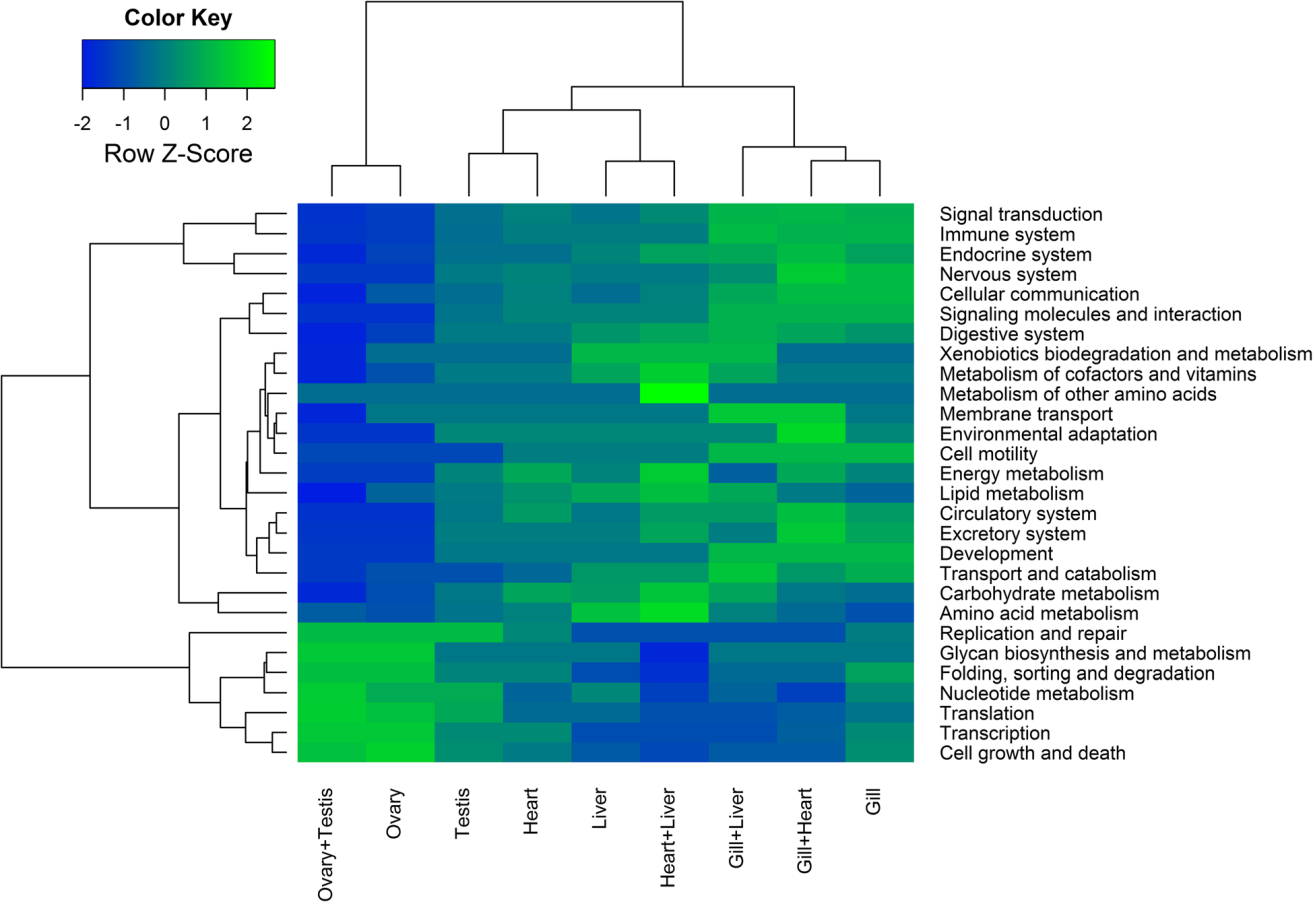
Heatmap showing contrasts between *T. thynnus* tissues and their combinations based on KEGG functional groups. Two-dimensional hierarchical clustering was performed on the difference between the counts of up- and down- regulated pathways within each KEGG functional category after GAGE gene set enrichment analyses. The analyses was conducted for each selected tissue and their combinations against other samples. Each tissue and their most informative pair-wise combinations are shown

Functional contrasts, inferred through KEGG pathway categorisation into respective functional groups (Fig. 7), were strongest between gill and ovarian tissue, also reflecting the fact that the greatest number of up- and down-regulated pathways was found for these tissues respectively. For the full list of pathways for each tissue and their pairwise combinations see Additional file 6. Gills were characterised by the largest diversity of functional categories, with immune system and signal transduction being the most highly represented, followed by nervous, endocrine, digestive, excretory system and cellular communication *inter alia*. This is consistent with the multi-purpose role of the fish branchial tissue in maintaining systemic homeostasis. The gill provides a key barrier to the outer aquatic environment and serves as the major site of respiratory gas exchange, pH and ion balance, nitrogenous waste excretion and immune defence, being controlled and co-ordinated by a complex web of neural and endocrine pathways [59, 60]. Tuna gills in particular have an unusually large surface area and thin blood-water barrier in respect to other active teleosts such as rainbow trout [61] permitting extremely efficient oxygen extraction. It is considered that this entails a higher metabolic cost with respect to the maintenance of water and ion balance, explaining in part the increased metabolic rates of tunas [3, 62]. As a representative of gill-specific processes, aquaporin 3b (*aqp3b*), a member of major intrinsic channel-forming proteins involved in teleost osmoregulation, water balance and nitrogen excretion [63], was confirmed in gill-specific Cluster 4 (Fig. 6).

In contrast to the gills, the ovaries showed over-representation of glycan biosynthesis and metabolism, folding, sorting and degradation, as well as cell growth and death, nucleotide metabolism, transcription, translation, replication and repair (Fig. 7). Primary oocyte growth is characterised by intense transcriptional activity and the presence of abundant ribosomes in highly granulated ooplasm [64, 65]. Maternal factors, protein and RNA species, are produced at this time and serve to support embryo development until the zygotic genome is activated [64]. Our analyses also suggest these processes are coupled with RNA degradation and mRNA surveillance pathways, crucial for maintenance of the integrity of transcriptional activity. Expression of a pescadillo (*pes*) homologue, important for early embryonic development in zebrafish [66], cell cycle progression and 60S ribosomal subunit synthesis [67, 68], and a transcript corresponding to exosome component 2 (*exosc2*) of the exosomal complex, comprising several 3′-5′ exoribonucleases involved in mRNA, 5.8S rRNA and small RNA processing [69], were confirmed as ovary-specific *cf*. other tested tissues (Fig. 6). These processes could also reflect the fact that the specific total RNA population of *T. thynnus* ovary seems to be dominated by the 5S RNA fraction, thus resembling the oocyte RNA complement of African clawed frog *Xenopus laevis* [28], Iberian ribbed newt *Pleurodeles waltl* [29] and a number of teleost species [30, 31]. It is believed that the 5S fraction is stored with aminoacyl-tRNAs in 7S and/or 42S ribonucleoprotein particles in the ooplasm and is progressively transferred, starting with vitellogenesis, into ribosomes by the end of oocyte differentiation [28]. This observation concurs well with our samples being taken during winter when the most advanced oocyte stage found in *T. thynnus* ovaries is perinucleolar [70]. Other typical ovarian transcripts were also present in ovary-specific Cluster 1 (see Additional file 3), including the aquaporin 1a homologue, previously detected in *T. thynnus* ovaries [22, 23] and involved in the process of oocyte hydration prior to ovulation, which is necessary for providing buoyancy in pelagic fish eggs [64, 71]. A major driving force of this water influx is the osmotic pressure produced by oocyte yolk protein cleavage by lysosomal proteases termed cathepsins. Cathepsins B, D and L have been implicated in this process in fish [64, 72]. Gardner *et al.* [23] found transcripts similar to cathepsin S, L and cathepsin Z precursor differentially expressed in ovarian relative to testis tissue in *T. thynnus* and postulated that the species likely uses different cathepsin proteases for this purpose. In this study, expression profiles of different cathepsin-like transcripts (A, L, F, S, Z) were detected across tissue clusters (see Additional file 3) with transcripts similar to cathepsin A and S placed in the ovary-specific Cluster 1, thus supporting this hypothesis.

Testes displayed a number of transcripts in common with ovaries leading to a separate, shared “gonad-specific” cluster using Biolayout *Express*^3D^ (Cluster 5). This cluster was characterised by the presence of transcripts generally important for germline development. These included a subunit of mediator complex (*med6*), which is a transcriptional co-activator of RNA polymerase II that is particularly important for expression of developmentally regulated genes [73]. Fanconi anaemia complementation group 1 (*fanci*) was also present, part of the DNA repair mechanism employed during homologous recombination in meiosis [74], as well as Piwi-like 2 (*piwil2*) homologue, essential for the germline integrity via transposable element repression and gonad development [75]. Analysis of ovary- and testis-associated transcripts as a single amalgamated group (ovary + testis) did not reveal any new additional pathways relative to separate analysis of the two tissues (see Additional file 6). A further Biolayout cluster comprised largely testis-specific transcripts (Cluster 6). Testis-associated pathways (Fig. 7) were more similar to those of other inspected tissues than to ovaries, however there was an overlap. Shared pathways were associated with nucleotide metabolism, replication, repair and translation. Some of the transcriptional differences between ovaries and testes have been identified for *T. thynnus* [23]. A similar transcript to one found differentially expressed in this previous study, was present here in the testis-specific Cluster 6; a component of t-complex *tcte1*, important for molecular interaction between sperm and egg *zona pellucida* [76]. This was also identified as testis-specific in rainbow trout by Rolland *et al.*, when compared to ovary, liver, muscle, gill and brain transcripts [77]. Intestinal fatty acid binding protein 2 (*fabp2*), suggested to play a role in sex dependent energy balance in *T. thynnus* [23], was found in heart-specific Cluster 2 (see Additional file 3) with its expression markedly elevated in testes compared to ovaries. The presence of several typically pituitary hormone transcripts, growth hormone (*gh*) and somatolactin (*sl*), were also apparent in the testis-specific cluster. The expression profile for somatolactin was re-investigated with qPCR and joint analysis of both datasets statistically confirmed (*p* < 0.05) this observation (Fig. 6). Both hormones are members of the growth hormone/prolactin family and are implicated in pleiotropic functions such as somatic growth, osmoregulation, lipid metabolism and reproduction [78, 79]. Extra-pituitary expression of growth hormone has been demonstrated in mammals and chicken testes [80], as well as fish [81] being localised to spermatogonia and primary spermatocytes in chicken and Sertoli cells surrounding the germ cells in Japanese eel. It has a paracrine role during spermatogenesis, stimulating proliferation of spermatogonia. This mitotic phase of spermatogenesis occurs in Mediterranean *T. thynnus* between October and January [82], corresponding to the collection period for our samples. Accordingly, a germ cell line marker vasa homologue (*vasa*), a putative RNA helicase found to be expressed in Pacific bluefin tuna spermatogonia [83], was also found to be up-regulated in testes of *T. thynnus* examined here. Primarily expressed in the pituitary gland, somatolactin mRNA has been detected across various tissues, including gonads in fish [84, 85]. These findings suggest *T. thynnus* testes actively transcribe *gh* and *sl*, warranting further investigation, especially in the context of reproductive cycle in both gonads.

During their spawning migrations, tuna utilise perigonadal mesenteric fat deposits as a source of metabolic energy for reproductive maturation and active swimming, which are primarily processed by the liver [82, 86]. In this study the *T. thynnus* liver was functionally characterised by lipid, amino acid and carbohydrate metabolism, fat digestion and absorption, bile secretion, metabolism of cofactors and vitamins, xenobiotic biodegradation and immune system (Fig. 7), consistent with the basic metabolic role of this organ [87] and agreeing with previous findings for the liver of *T. thynnus* [22]. Among the transcripts showing predominant expression in the liver, members of a haem-containing superfamily of cytochrome P450 mono-oxygenases, involved in metabolic biotransformation of xenobiotics and lipids often associated with liver [88], were found (see Additional file 3). The liver is also a key production site for coagulation and complement proteolytic cascade components, mediators of blood clotting and elements of the innate immune system. Two representative transcripts, homologues of blood coagulation factor V (*f5*), pivotal proteins in maintaining haemostasis with dual pro-coagulant and anti-coagulant activities in humans [89], and complement component C4 (*c4*), which plays a crucial role in the classical and lectin pathways of complement activation as a subunit of the C3 convertase [90], were found to be significantly up-regulated in the liver compared to other tissues (Fig. 6).

Tuna cardiac function has been the subject of extensive investigation, as the heart operates at ambient temperature while supporting high metabolic rates and endothermic physiology [4]. Tuna have large hearts that function at close to maximum stroke volumes and depend, more than other teleosts, on oxygen consuming fatty acid oxidation as a fuel source, similar to mammals [62, 91]. Mitochondria isolated from skipjack tuna ventricles have a higher capacity to oxidise lactate than other metabolites, suggesting that lactate is also an alternative fuel source, especially during high intensity swimming when blood lactate is elevated [92]. These processes were well represented in the pathways and functional categories assigned to *T. thynnus* heart (*apex ventriculi*) compared to gill, liver and gonads in this study. Lipid (fatty acid degradation), energy (oxidative phosphorylation), carbohydrate metabolism (pyruvate metabolism, glycolysis/gluconeogenesis) and circulatory system (cardiac muscle contraction) were most prominent categories in functional contrasts displayed for the heart in Fig. 7. The ability to maintain cardiac performance during temperature acclimation, especially to cold, has been linked at the transcriptomic and functional level to Ca^2+^-dependent excitation-contraction coupling (EC) in *T. orientalis* [24, 93]. Up-regulation of sarcoplasmic reticulum calcium-ATPase 2 (*atp2a2a* or *serca2*) homologue, vital for Ca^2+^ removal from cytosol into sarcoplasmic reticulum in vertebrates [94], has been identified in *T. thynnus* heart-specific Cluster 2 (Fig. 6 and see Additional file 3).

Amalgamating transcripts by pairs of tissues (*e*.g. gill + liver, gill + heart, gill + testis *etc*.), in order to resolve shared and unique pathways, showed pairings which included gill, heart and liver to be most informative and revealed functional signatures shared between these tissues. For instance, the most pronounced feature in Fig. 7 is the up-regulation of metabolism of other amino acids, β-alanine metabolism specifically (see Additional file 6), found exclusively for the heart + liver combination. β-Alanine is produced in vertebrate liver and is used as a rate-limiting precursor for synthesis of histidine-containing dipeptides such as carnosine and anserine in skeletal muscle, heart and brain [95, 96]. These compounds are involved in intracellular pH buffering by tissues exhibiting high dependence on anaerobic glycolytic metabolism in which lactic acid is generated, such as fast-twich white skeletal muscle in fish [95]. High levels of histidine-containing dipeptides have been recorded for skipjack tuna white muscle, with anserine largely exceeding carnosine [97]. Aside from strong buffering capacity, carnosine has also been found to exhibit antioxidant, pro-contractile, chelating and anti-glycating properties and it is considered that β-alanine diet supplementation can have protective cardiac effects due to increased levels of carnosine [96]. Other important pathways mediating environmental and physiological adaptation were also identified. HIF-1 (hypoxia inducible factor) signalling pathway was found to be up-regulated in gill + heart and heart + liver combinations. Hif is a transcriptional factor governing the physiological response to hypoxia that induces a wide range of changes in energy metabolism, red blood cell formation, vascularisation, oxygen transport and apoptosis [98]. Regulation of *hif-1α* has been reported in haemaotopoietic and gas-exchange organs in *T. orientalis* as a result of cold temperature acclimation [99]. ABC transporter systems were found to be up-regulated in gill + heart and gill + liver combinations (see Additional file 6). The ATP-binding cassette (ABC-transporters) are trans-membrane spanning proteins involved in substrate translocation across membranes and form the basis of multi-xenobiotic resistance mechanism in aquatic animals, with high detoxification relevance. They also participate in transport of lipids, ions and other metabolites and have been attributed various tissue-specific roles [100]. Finally, insulin signalling pathway was found common to all pairwise combinations of gill, heart and liver (see Additional file 6). Exerting its effect through insulin receptors and associated effector signalling cascade actively transcribed in various peripheral organs, insulin has been implicated in wide range of processes in fish, including vital carbohydrate and lipid metabolism, appetite regulation, growth and development [101].

## Conclusions

Sequence data generated from a novel mixed-tissue *T. thynnus* cDNA library provides an important transcriptomic resource that can be further employed for study of various aspects of *T. thynnus* ecology and genomics, with strong applications in aquaculture. We have developed and validated a new and flexible gene expression profiling tool, in the form of a 15 K oligonucleotide DNA microarray, which produced biologically relevant information agreeing with prior observations made in the literature, providing external validation for our new resource. The resolved expression profiles of gill, heart, liver and gonads provide a useful starting point for exploring gene expression patterns in *T. thynnus* and for planning new transcriptomic studies. The oligo-microarray can also be further expanded to include un-annotated transcripts from the generated library with the aim of extending their functional characterisation through correlation with annotated entities. It is important to keep in mind that even tissue specific profiles can be population dependent [102], warranting further research on captive and wild tuna populations.

### Availability of supporting data

The raw sequence data have been submitted to the EBI Sequence Read Archive (SRA) study PRJEB7253 (http://www.ebi.ac.uk/ena). The microarray design and data are available in the EBI ArrayExpress database (www.ebi.ac.uk/arrayexpress) under accession numbers A-MTAB-553 and E-MTAB-3412, respectively.

## Additional files

Additional file 1:
**Mapping of** ***T. thynnus*** **transcripts to** ***O. niloticus*** **genome.** (TXT 2853 kb) Additional file 2:
**Mapping of** ***T. thynnus*** **transcripts to** ***T. orientalis*** **genome.** (TXT 5528 kb) Additional file 3:
**Results of network analyses and clustering using Markov algorithm for** ***T. thynnus*** **transcripts.** Log2 normalised averaged signals are provided for each probe. (XLSX 904 kb) Additional file 4:
**RT-qPCR related data. Table sheets containing primer sequences for genes included in qPCR analyses, BestKeeper stability for reference genes and Log2 transformed data for all targets.** (XLSX 25 kb) Additional file 5:
**Kruskal-Wallis test statistic for combined expression profiles of target genes based on microarray and qPCR data.** (XLSX 14 kb) Additional file 6:
**KEGG pathway analyses of** ***T. thynnus*** **tissue expression profiles.** The analyses were performed by choosing each tissue as experimental state and all other as reference. The same was repeated by denoting combinations of two tissues together as a test sample. Data are given only for statistically significant pathways (q < 0.1) and the sign of stat. mean value indicates the direction of regulation. (XLSX 51 kb)
